# Stock assessment and end-to-end ecosystem models alter dynamics of fisheries data

**DOI:** 10.1371/journal.pone.0171644

**Published:** 2017-02-15

**Authors:** Laura S. Storch, Sarah M. Glaser, Hao Ye, Andrew A. Rosenberg

**Affiliations:** 1 Department of Mathematics and Statistics, University of New Hampshire, Durham, New Hampshire, United States; 2 Korbel School of International Studies, University of Denver, Denver, Colorado, United States; 3 Secure Fisheries, One Earth Future Foundation, Broomfield, Colorado, United States; 4 Scripps Institution of Oceanography, University of California, San Diego, La Jolla, California, United States; 5 Union of Concerned Scientists, Cambridge, Massachusetts, United States; Technical University of Denmark, DENMARK

## Abstract

Although all models are simplified approximations of reality, they remain useful tools for understanding, predicting, and managing populations and ecosystems. However, a model’s utility is contingent on its suitability for a given task. Here, we examine two model types: single-species fishery stock assessment and multispecies marine ecosystem models. Both are efforts to predict trajectories of populations and ecosystems to inform fisheries management and conceptual understanding. However, many of these ecosystems exhibit nonlinear dynamics, which may not be represented in the models. As a result, model outputs may underestimate variability and overestimate stability. Using nonlinear forecasting methods, we compare predictability and nonlinearity of model outputs against model inputs using data and models for the California Current System. Compared with model inputs, time series of model-processed outputs show more predictability but a higher prevalence of linearity, suggesting that the models misrepresent the actual predictability of the modeled systems. Thus, caution is warranted: using such models for management or scenario exploration may produce unforeseen consequences, especially in the context of unknown future impacts.

## Introduction

Over the last several decades, global fish populations have suffered from detrimental decreases in abundance, with one in four fisheries experiencing collapse [1–3]. The current US fisheries modeling framework utilizes single-species stock assessment models to inform management decisions about commercially relevant species. With the collapse of so many fisheries, there has been a growing realization of the importance of biodiversity and species-to-species interactions, which may play important roles in maintaining robustness of exploited ecosystems [4–7]. To better understand multi-species effects, the current single-species framework can be extended to include species that play important ecosystem roles, but may be of little commercial interest. Ecosystem models such as Atlantis implement this by modeling multiple species and functional groups and their interactions with physical environmental factors and human pressures on the environment. Although ecosystem models were previously used mainly as conceptual tools [8], their applications have expanded to include, e.g., analyzing the impact of different fishing scenarios [9] and management strategy evaluation [10], and they are expected to be used directly for management in the next several years [11].

As application of ecosystem models expands rapidly, validation of model outputs via a variety of methods will increase the robustness of such applications. Several metrics have been developed to quantifiably validate ecosystem model outputs [12] and these metrics have recently been applied to the Atlantis model [13]. Model assessment takes the form of comparing model output with historical data. The ability of the output to match the data is assessed, for example, through correlation coefficients and error estimates. While these assessments are necessary steps in the model validation process, they may not necessarily provide a satisfactory indication of whether or not a model is sufficient for its intended application.

Here, we introduce an additional method of validation that addresses to what extent model outputs are preserving dynamical signal of the input data. Few attempts have been made to determine whether ecosystem models preserve underlying system dynamics even though such properties can be critical when models are applied for management purposes [14]. By necessity, an ecosystem model is a simplified approximation of the complex systems that exist in nature. However, the simplifications implemented in any model can dramatically affect model output [15, 16]. We assess the extent to which model outputs retain the dynamical signatures of model input data via two characteristics: nonlinearity and predictability. Both of these measures can give some indication for how the assumed behavior within the model differs from the behavior of the population or ecosystem of interest. In this study, we compare the prevalence of nonlinear signals and predictability in model outputs to those in model input data in order to quantify how fisheries and end-to-end ecosystem models may alter the dynamical signatures of fisheries data.

Detecting nonlinearity in natural systems, and comparing this with the presence of nonlinearity in model outputs, is an important way to quantify the differences between system dynamics and model dynamics. Although nonlinearity in natural systems is well documented (e.g., [17, 18]), it is not often incorporated into modeling approaches. Linear approximations of nonlinear systems can be reliable for short-term forecasting, but they may overestimate the long-term stability and predictability of nonlinear systems [19]. This overestimation of population stability can lead to overconfidence in forecasting abilities, which affects management decisions. Secondly, nonlinearity and high variability are frequently encountered together [20, 21], suggesting that achieving sustainable population levels may be more difficult when nonlinear dynamics are at play. Finally, the limited time horizon for accurate future predictions of nonlinear systems must be considered [22]. In general, accuracy and precision decrease as predictions extend further into the future [17], due to a confluence of process, model, or measurement errors. Therefore, accurate long-term predictions of complex systems may not be possible.

We also use predictability, a characteristic of a given time series, to measure how input data and model outputs may differ. Here, predictability is defined as how well future values can be forecasted based on past and present conditions. In the context of time series forecasting models, predictability also measures how deterministic a system is / observation noise in the system [23]. We can evaluate the determinism of the system because the deterministic trajectory of a dataset (the noise-free trajectory, guided only by the system dynamics) is predictable. On the other hand, the more observational noise present in a data set, the less predictable it will be, as the deterministic dynamical signal is confounded with random noise. Because traditional modeling approaches tend to be parametric (and also implicitly assume dynamical stationarity), we expect model outputs to display high levels of predictability. These high levels of predictability may not necessarily reflect the predictability of the modeled system, however.

In this study we evaluate whether the dynamics contained in fisheries data differ from the dynamics of fisheries model outputs. We compare fisheries time series comprised of two types of collected data: (1) California commercial fish landings and (2) California Cooperative Oceanic Fisheries Investigations (CalCOFI) ichthyoplankton abundance surveys; and two types of model outputs: (1) estimates of population abundance from single-species stock assessment models and (2) estimates of abundance for both single-species and aggregated functional groups from the ecosystem-based California Current Atlantis Model (CCAM). The stock assessment models use both landings and survey abundances as input, while the Atlantis model uses the output from stock assessment models as input.

**Fig 1:**
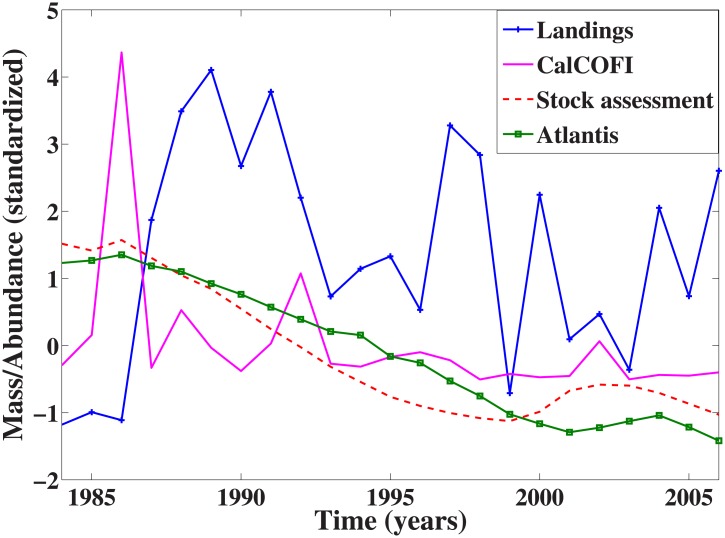
Comparison of four time series for Pacific hake. Comparison of landings, CalCOFI, stock assessment, and Atlantis time series for Pacific hake. Landings data are in the form of yearly landings at the dock, CalCOFI data are yearly larval abundance, and stock assessment and Atlantis data are yearly biomass estimates.

We hypothesize that model outputs will have predominantly linear signals and higher predictability than model inputs due to myriad model estimations, simplifications, aggregation of species, and model assumptions of fundamentally stable system dynamics. To show the different time series types, Fig 1 compares time series of observed data and model outputs for *Merluccius productus* (Pacific hake). Variability in both model outputs is greatly reduced in comparison to both data types. This is not unexpected; in fact, model smoothing of variability is a feature, not a bug, of the modeling process. But the supposition tested here is that this smoothing process removes some of the critical dynamic information contained in the raw data. While the greater range of variability in the two observed data series is likely due to convolved observational error, it can also reflect deterministic (i.e., predictable) nonlinear dynamics. Because of the presence of observational error, we predict decreased prediction skill for the two data types as compared to model output. However, because the methodology we use distinguishes nonlinear signals from random noise [17], we expect that this observational error is unlikely to effect our ability to detect nonlinearity. Indeed, given the well-known occurrence of nonlinear signals in fisheries data [18, 19], we expect that the fisheries data and fisheries model outputs will not be in agreement, suggesting a loss of dynamical signal as a side effect of the model processing.

## Materials and methods

### Data

Time series of California commercial landings data, CalCOFI ichthyoplankton abundance survey data, stock assessment output, and CCAM output were analyzed using nonlinear forecasting methods. Table 1 summarizes the four different types and their duration. All time series are standardized and first differenced. We use first differencing to remove first-order autocorrelation and obtain more conservative estimates of forecasting ability. For both landings and stock assessment data, the early years were trimmed so that the resulting time series best captured a period during which the fishery was fully developed (i.e., so that the time series reflect the dynamics of underlying biomass changes instead of changes in fishing effort). CalCOFI ichthyoplankton abundance data were obtained from the Southwest Fisheries Science Center and span 1951–2007. The raw abundance data are in the form of space- and time-averaged (yearly) larvae per square meter, and combined to produce one time series per species following [24]. Additional information on CalCOFI data processing can be found on the CalCOFI website (http://calcofi.org/data.html). Landings data were obtained from the California Department of Fish and Game (https://www.dfg.ca.gov/marine/groundfishcentral/comdata.asp) and are total annual landings summed over all California ports. Stock assessment outputs were obtained from published reports, available from the Pacific Fishery Management Council (www.pcouncil.org). For each stock, a single time series was created from yearly biomass data by aggregating across all age classes when available, and using spawning stock biomass otherwise. Nearly all U.S. west coast stock assessment reports employ the Stock Synthesis model [25]. CCAM outputs were obtained from Isaac Kaplan at the Northwest Fisheries Science Center and span 1950–2008. CCAM output is in the form of yearly age-aggregated biomass estimates for 13 individual species and 46 functional groups for a total of 59 time series [26, 27].

**Table 1:** Available years (prior to trimming).

Data/Model*	Type	# Time series	Years available
**Data**	Landings	49	1928–2006
**Data**	Abundance survey	23	1951–2007
**Model output**	Stock assessment	36	1892–2009
**Model output**	Atlantis	59	1950–2008

* Whether the time series is data or processed model output.

Exploitation status affects the likelihood of detecting nonlinear dynamics in time series [19]. Thus, to facilitate an unbiased comparison, we restrict our analysis to mostly species that are subject to exploitation. Among the species in the CalCOFI dataset, we use only time series from exploited species. By definition, landings data and stock assessments only come from exploited species. Finally, the majority of species in the Atlantis model are exploited, except for several Atlantis functional groups that contain unexploited species. Landings data, stock assessment, and Atlantis outputs have a high degree of overlap, but show less overlap with CalCOFI data. For example, all 36 stock assessments used here have associated landings data but only 10 have ichthyoplankton abundance estimates from the CalCOFI surveys.

To distinguish the role of observational error in detecting nonlinearity and quantifying predictability in our time series, we test the effects of adding observational error on the dynamics of Atlantis and stock assessment model output. For each time series, independent and identically distributed (i.i.d.) error, drawn from a normal distribution with standard deviation 0.3, is added (note that the time series are standardized beforehand). The nonlinear analysis is then repeated for 100 realizations of each time series.

### Forecasting model

**Fig 2:**
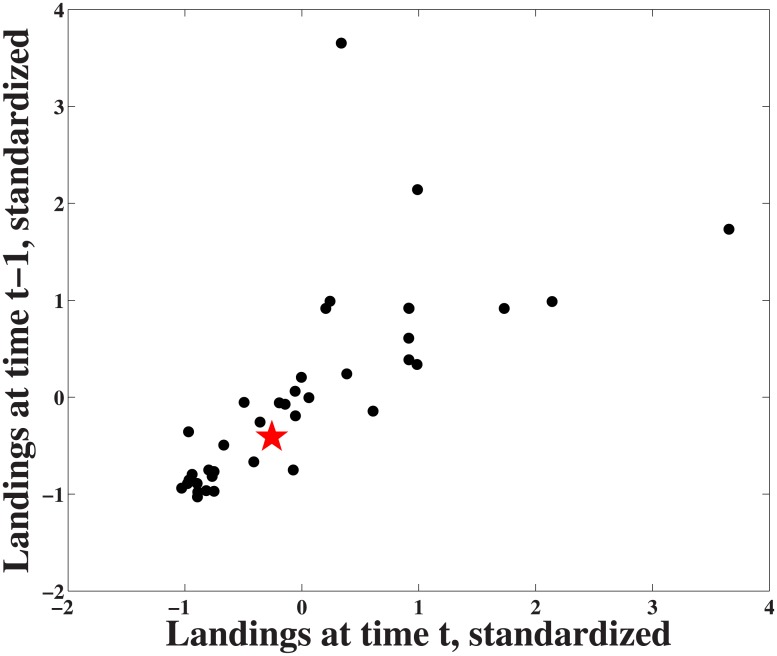
Time lagged coordinate system. Two-dimensional representation of time lagged coordinates for standardized sablefish landings, where axes are time series at time (t-1) and time (t). The red star represents a predictee (here, a vector of length 2). The E+1 (i.e. 3) nearest neighbors will be used to make a forecast for the predictee at time t+1.

The nonlinear forecasting method employed here includes two steps: simplex projection and S-map [17, 23]. These methods evaluate predictive skill and nonlinearity based on Takens’ theorem of lagged coordinates [28]. In essence, this approach uses information in the dynamics of past observations (i.e., lags) to make short-term forecasts, and is particularly useful when the equations driving the system are unknown. Fig 2 depicts this process, using a two-dimensional lagged coordinate system, where standardized sablefish (*Anoplopoma fimbria*) landings at time t-1 are plotted against the standardized landings at time t. The star on the graph represents a specific predictee (the current point). To predict the following year, the nearest neighbors (i.e., closest data points in terms of Euclidean distance) of the predictee are used. The number of lagged coordinates used to define the space is the embedding dimension, E.

Following [19], we use simplex projection to identify the optimal embedding dimension for each time series. Here, we select the value of E that results in the best possible prediction (i.e., the lowest error and highest accuracy calculated from observed and predicted points). This is accomplished by dividing the time series into a library set and a predictive set, with the library set forming the model that is used to forecast the predictive set. The ability of the model to forecast the predictive set quantifies model accuracy [29]. Here, the library set was composed of all but one vector in the time series, and the predictive set contained the withheld vector (leave-one-out cross-validation). Thus, each point is predicted using a model trained on the set of data excluding that point. This process was repeated for each vector in the time series so that all predictions were made out-of-sample.

From the model-produced forecasts, we calculated rho (correlation coefficient between observed and forecast values), MAE (mean absolute error), and RMSE (root mean squared error) to assess accuracy. P-values were computed for the significance of rho > 0 via Fisher’s z-transformation to provide objective criteria for selecting time series with clear predictive dynamics. Modeled time series with rho p-values ≥ 0.05 were eliminated from further analysis; such series do not provide a reliable signal for our purposes (they may be too short or too noisy).

After choosing the best E, we evaluate the presence of nonlinearity using the S-map procedure (short for “sequentially locally weighted global linear map”) [23]. Unlike simplex projection, where E+1 nearest neighbors are used to make a prediction, all library points are included in the S-map prediction and are weighted depending on their proximity to the predictee. These weight are controlled by a tuning parameter, theta. When theta is equal to zero, all neighbors receive equal weight in the forecast; for larger values of theta, closer points are more heavily weighted than distant points (i.e., as theta increases, the model becomes more nonlinear).

To quantify nonlinear signals in our data, we compared model error (mean absolute error) in S-map for parameterizations where theta = 0 to parameterizations where theta was greater than 0. If the nonlinear parameterization resulted in significantly reduced error (determined by a randomization test; see [30]), the dynamics in the model were classified nonlinear.

A detailed explanation of simplex projection and s-map can be found in [17–19, 23, 29, 31]. Information about the mathematical theory behind the methodology can be found in the supplemental material (S1 File). Additionally, an R package containing these methods is available from the Comprehensive R Archive Network (https://cran.r-project.org/web/packages/rEDM/index.html).

## Results

Results are presented for standardized first differenced times series. The six categories of time series are:

1. Landings—California commercial landings data for individual species
2. Abundance Survey—CalCOFI ichthyoplankton yearly abundance survey data for individual species
3. Stock Assessment—Stock assessment model output in biomass for individual species
4. Atlantis—Atlantis model output in biomass for individual species and functional groups
5. Stock Assessment + random error—Stock assessment model output with added random error
6. Atlantis + random error—Atlantis model output with added random error

Results for individual species are available in S1 Table (and for completeness, embedding dimension results are available in S2 Table). Both forms of observed data [(1) and (2)] had lower prediction skill than the noise-free model outputs [(3) and (4)]. Predictability results are presented in Table 2. Landings data were significantly less predictable than both types of model output (p < 0.0001, Tukey’s HSD). The difference between CalCOFI and Atlantis was significant (p < 0.0001, Tukey’s HSD), but the difference between CalCOFI and stock assessment time series was not.

**Table 2:** Average (+/- SD) prediction skill (rho) for s-map model.

Data/Model	Type	# Time series	Average rho
**Data**	Landings	49	0.259±0.212
**Data**	Abundance survey	23	0.559±0.151
**Model output**	Stock assessment	36	0.667±0.263
**Model output**	Stock as. + noise	3600	0.447±0.195
**Model output**	Atlantis	59	0.862±0.154
**Model output**	Atlantis + noise	5900	0.579±0.117

As expected, observed data had higher prevalence of nonlinearity than model outputs: the likelihood of nonlinear classification was significantly higher in landings than in model outputs (logistic regression, Wald test, p = 0.0026, Table 3) and significantly higher in CalCOFI time series than in model outputs (p = 0.0311, Table 3). Among the datasets, 22 of 49 landings, 2 of 23 CalCOFI, and 3 of 36 stock assessment time series did not meet our criteria of significant forecast skill (p < 0.05) and were therefore excluded from the nonlinear analysis.

**Table 3:** Presence of nonlinearity in each data type.

Data/Model	Type	# Time series	% Nonlinear
**Data**	Landings	27	59%
**Data**	Abundance survey	21	43%
**Model output**	Stock assessment	33	18%
**Model output**	Stock as. + noise	3019	18%
**Model output**	Atlantis	59	15%
**Model output**	Atlantis + noise	5871	14%

We test the nonlinearity and predictability of model outputs with added random noise to determine if the higher prevalence of nonlinearity in the landings and CalCOFI datasets is an artefact of random error in the unprocessed data being filtered out in the fisheries and ecosystem models. If this were the case, the results for the model outputs with added random noise should resemble the results for the two forms of raw data. The addition of random noise resulted in significantly reduced predictability for both Atlantis and stock assessment model outputs (Tukey’s HSD, both p < 0.0001) but had no effect on the prevalence of nonlinearity, indicating that the models are, indeed, altering the inherent dynamics present in landings and abundance time series.

Time series length can affect the inferred dimensionality, predictability, and nonlinearity; shorter time series are expected to show lower prediction skill due to a lack of resolved signal. For stock assessment and landings data, series were cropped to best reflect the time period when the fishery was active, resulting in different lengths. The average length of a landings time series with significant forecast skill (p < 0.05) was approximately 60 ± 20 years (standard deviation), and there was no significant difference found between the lengths of the 16 nonlinear and 11 linear time series (60 ± 20 versus 59 ± 20, p = 0.81, one-way ANOVA). The average length of a stock assessment time series was approximately 44 ± 12 years. No significant difference exists between lengths of 6 nonlinear versus 27 linear stock assessment time series (45 ± 9 versus 43 ± 13, p = 0.79, one-way ANOVA). Landings time series were significantly longer than the other data types (Tukey’s HSD, landings vs. Atlantis, p = 0.03, landings vs. CalCOFI and stock assessment, p < 0.0001).

## Discussion

In this study, the model output estimates of abundance showed lower degrees of nonlinearity and higher degrees of predictability compared to larval abundance observations. While higher predictability is an expected and even desired outcome, it is a misleading measure of actual system stability unless the model outputs are representative of true population abundances. Noise reduction is a goal of fisheries modeling, but the decrease in nonlinearity indicates that the models are not only reducing noise; they are removing dynamical signal. If the real population dynamics are nonlinear, a linearization of the dynamics will distort our understanding of the dynamical stability of the system and could invalidate conceptual understanding of the system gained from the model.

Similarly, landings time series have a much higher prevalence of nonlinearity and are far less predictable than the model output types. Although trends in landings do not necessarily reflect population abundance dynamics, we believe this discrepancy warrants further examination, as highly nonlinear data used as input for stock assessment and ecosystem models are yielding linearized model outputs. The layers of estimation contained in highly parameterized fisheries models are filtering out nonlinearity and uncertainty, and biasing our view of fisheries systems towards linearity and higher predictability. Such results are in agreement with previous studies finding that models of nonlinear time series can result in more stable dynamics than exist in reality [32].

As expected, adding noise to model output time series reduces predictability compared to the original data. However, the level of nonlinearity remained unchanged: noisy model outputs were just as nonlinear as the noise-free model outputs and significantly less nonlinear than landings or abundance time series. Thus, the increased predictability of model output (compared to model inputs) cannot be attributed simply to models successfully filtering out observational error. If that were the case, then adding noise to the model output data should produce a level of predictability and nonlinearity similar to that of model inputs (i.e., landings or abundance data). However, since predictability was the only property that changed, noise filtering cannot be the *only* processing that occurs to produce model outputs from model inputs. Instead, our results suggest that the models are creating smoothed, more easily predictable time series that do not retain the dynamical signals of the input data. In other words, while model outputs are more predictable, this predictability is not a real property of nature, but is artificially introduced by model equations.

Although the smoothing effects of parameterized models may be well-known among the ecosystem modeling community, end-users such as management officials or stakeholders will be less familiar and may misinterpret model outputs. As the use of ecosystem models proliferates and is applied to inform management and policy, education about appropriate usage and interpretation is critical and needs to be explicitly communicated. While Atlantis can be a helpful tool for illustrating the myriad effects a system might have in response to a given action, we caution against its use for fisheries forecasting (particularly long-term forecasting) since the true dynamics of the system are unknown. We additionally caution against the use of stock assessment model outputs as Atlantis model inputs, since we have shown that dynamical signal is lost in the stock assessment modeling process.

Recent standards for ecosystem model assessment have been developed by an expert panel [33] and include standards for how well model outputs match historical data, how well model parameters match parameters from literature or established theory, and general model behaviors such as persistence of functional groups over time. Such standards are an important step in critically evaluating ecosystem models as their usage becomes more common and more diverse. We propose incorporating the metrics tested in this study to the already existing standards. Comparing nonlinearity of model outputs versus historical data will give critical insights into system stability and highlight the difference between the dynamics of the model versus the dynamics of the modeled system.

For model usage in management decisions, we make several precautionary suggestions. First, the stability and high degrees of prediction skill achievable in the models is not indicative of nature’s stability nor of our ability to accurately predict it. Because the true stability of these systems is unknown, we suggest heightened precautions when setting catch limits and expansion of confidence intervals based on stock assessment models. Second, we suggest a constraint on how far into the future we rely on predictions, as the models can similarly overestimate the stability of fisheries systems. Third, model consideration of nonlinear dynamics, or at the minimum, increased population variability, would lead to management guidelines that better match the goals of species persistence and sustained ecosystem services, as scenario exploration that considers the presence of nonlinear dynamics would lead to a more realistic picture of the variety of dynamical responses possible for the system [34]. Finally, using complex models to manage complex systems does not lead to better understanding of the system [35]. As model complexity increases, so do myriad types of model error [36]. There likely exists a fundamental limitation in ability to model real-world systems with complex models, and consequently, simpler management rules or indicators could result in more robust management.

While we use these models to inform management, it is critical to understand fully how the models are interpreting the system dynamics; not only to guide our own estimates of uncertainty, but also for communicating that uncertainty to policy makers. We present one method for quantifying the differences between data and model outputs, and the resulting illusion of predictability that results from stock assessments and the Atlantis ecosystem model. Future model assessments should incorporate similar metrics for understanding changes in data signals to promote development of the best possible science for fisheries management. Improvements in understanding ecosystem dynamics and the effect of data processing would aid in improving the validity of model forecasts.

## Supporting information

S1 Table Nonlinearity and predictability results for all species individually across the four time series types. Species with p rho > 0.05 (or negative max rho) were excluded from the nonlinearity results in Table 3 of the paper.
(DOCX)

S2 Table Embedding dimension (E) results for each data type. (DOCX)

S1 File Mathematical theory of the study’s methodology. (DOCX)

S1 Fig Lorenz attractor in X-Y-Z coordinates, σ = 10, b = 8/3, r = 26. (EPS)
