# Supplementary material for: Stock assessment and end-to-end ecosystem models alter dynamics of fisheries data
Source: PLoS One. 2017 Feb 15;12(2):e0171644. doi: 10.1371/journal.pone.0171644 (PMC5310756; doi:10.1371/journal.pone.0171644)
Supplement: S1 Table — Species with p rho > 0.05 (or negative max rho) were excluded from the nonlinearity results in Table 3 of the paper. (DOCX) [file pone.0171644.s001.docx]

**Supplementary material**

Table S1. Nonlinearity and predictability results for all species individually across the four time series types. Species with p rho > 0.05 (or negative max rho) were excluded from the nonlinearity results in Table 3 of the paper.

| Type | Species | Max rho | p rho | Nonlinear (Yes/No) |
| --- | --- | --- | --- | --- |
| Landings | Abalone | 0.25472 | 0.020142444 | N |
| Landings | Albacore | 0.21932 | 0.05229047 | N |
| Landings | Anchovy | 0.36466 | 0.002694409 | Y |
| Landings | Barracuda | -0.052814 | 0.334982282 | Y |
| Landings | Bay shrimp | 0.58588 | 0.000394183 | Y |
| Landings | Bluefin | 0.34659 | 0.001242948 | N |
| Landings | Bonito | 0.25274 | 0.014748063 | N |
| Landings | Cabezon | 0.26077 | 0.016361104 | Y |
| Landings | Clams | 0.10858 | 0.278589943 | N |
| Landings | Cucumbers | 0.2402 | 0.120014712 | N |
| Landings | Dungeness crab | 0.1577 | 0.090124845 | Y |
| Landings | Flounder | 0.405 | 0.000266428 | Y |
| Landings | Flying fish | 0.57566 | 4.59E-07 | Y |
| Landings | Giant sea bass | 0.25156 | 0.017677447 | N |
| Landings | Grenadier | -0.15462 | 0.302591043 | N |
| Landings | Hake | 0.18609 | 0.205905679 | N |
| Landings | Halibut | 0.071206 | 0.273916808 | N |
| Landings | Herring | 0.17501 | 0.183618399 | N |
| Landings | Jack mackerel | 0.29104 | 0.01795633 | N |
| Landings | Lingcod | 0.30752 | 0.00312989 | Y |
| Landings | Lobster | 0.068498 | 0.277539599 | N |
| Landings | Loligo | 0.081968 | 0.321064671 | N |
| Landings | Ocean shrimp | 0.38719 | 0.01042279 | N |
| Landings | Ocean whitefish | 0.44726 | 1.74E-05 | Y |
| Landings | Octopus | 0.14302 | 0.11413033 | Y |
| Landings | Pacific mackerel | 0.28889 | 0.006755385 | N |
| Landings | Perch | 0.48355 | 2.83E-06 | Y |
| Landings | Prawns | 0.66987 | 0.000144649 | Y |
| Landings | Rock bass | 0.40939 | 0.032516451 | N |
| Landings | Rock crab | 0.18648 | 0.167993899 | N |
| Landings | Rockfish | 0.010537 | 0.465632946 | Y |
| Landings | Sablefish | 0.4958 | 0.000761106 | Y |
| Landings | Salmon | 0.40007 | 0.000317463 | Y |
| Landings | Sanddab | 0.096817 | 0.201727041 | N |
| Landings | Sardine | 0.191 | 0.051613629 | Y |
| Landings | Scorpionfish | 0.27968 | 0.00738199 | N |
| Landings | Sharks | 0.060716 | 0.312027717 | Y |
| Landings | Sheephead | 0.60181 | 1.07E-09 | Y |
| Landings | Skate | -0.27084 | 0.00921268 | N |
| Landings | Skipjack | -0.24918 | 0.01860347 | Y |
| Landings | Smelts | 0.31524 | 0.002809731 | Y |
| Landings | Sole | 0.18016 | 0.062415651 | Y |
| Landings | Swordfish | 0.27958 | 0.008929476 | Y |
| Landings | Thornyhead | 0.27976 | 0.079554744 | N |
| Landings | Turbot | 0.35829 | 0.001704436 | N |
| Landings | Urchin | 0.35765 | 0.033390705 | N |
| Landings | White croaker | 0.39224 | 0.000239555 | Y |
| Landings | Yellowfin | 0.75164 | 6.27E-06 | Y |
| Landings | Yellowtail | 0.14944 | 0.10227313 | Y |
| CalCOFI | Aurora rockfish | 0.14816 | 0.237051511 | N |
| CalCOFI | Bigmouth flounder | 0.64605 | 5.06E-06 | Y |
| CalCOFI | Blacksmith | 0.55635 | 0.000156141 | N |
| CalCOFI | Bocaccio | 0.49507 | 0.002399433 | N |
| CalCOFI | Cabezon | 0.72803 | 3.20E-07 | Y |
| CalCOFI | California halibut | 0.52111 | 0.000929488 | N |
| CalCOFI | California tonguefish | 0.54603 | 0.000484235 | N |
| CalCOFI | Combfish | 0.54142 | 0.001219487 | N |
| CalCOFI | Croakers | 0.62285 | 7.49E-05 | Y |
| CalCOFI | Dover sole | 0.60245 | 5.21E-05 | N |
| CalCOFI | English sole | 0.64206 | 6.06E-06 | Y |
| CalCOFI | Hornyhead turbot | 0.69645 | 3.86E-07 | Y |
| CalCOFI | Jack mackerel | 0.5567 | 0.000235464 | Y |
| CalCOFI | Northern anchovy | 0.19919 | 0.156380698 | N |
| CalCOFI | Pacific barracuda | 0.74408 | 1.77E-08 | Y |
| CalCOFI | Pacific hake | 0.57069 | 0.000375921 | N |
| CalCOFI | Pacific mackerel | 0.60639 | 7.63E-05 | N |
| CalCOFI | Painted greenling | 0.45289 | 0.003274905 | Y |
| CalCOFI | Rockfishes | 0.60936 | 0.000200433 | N |
| CalCOFI | Sand bass | 0.68089 | 1.87E-06 | Y |
| CalCOFI | Sardine | 0.50229 | 0.001050984 | N |
| CalCOFI | Slender sole | 0.76589 | 1.83E-06 | N |
| CalCOFI | Thornyhead | 0.42672 | 0.007044492 | N |
| Stock assessment | Arrowhead flounder | 0.8012 | 1.02E-11 | N |
| Stock assessment | Black rockfish | 0.93052 | 2.22E-16 | N |
| Stock assessment | Blackgill rockfish | 0.64376 | 9.00E-05 | N |
| Stock assessment | Blue rockfish | 0.89959 | 0 | N |
| Stock assessment | Bocaccio | 0.90183 | 0 | N |
| Stock assessment | Cabezon (Northern California) | 0.51207 | 1.62E-05 | N |
| Stock assessment | Cabezon (Southern California) | 0.63869 | 4.28E-09 | N |
| Stock assessment | Cabezon CAS | 0.50871 | 3.08E-05 | N |
| Stock assessment | Canary rockfish | 0.72851 | 3.73E-09 | N |
| Stock assessment | Chilipepper rockfish | 0.46789 | 0.000881166 | N |
| Stock assessment | Cowcod | 0.84318 | 0 | N |
| Stock assessment | Darkblotched rockfish | 0.82156 | 7.91E-10 | N |
| Stock assessment | Dover sole | 0.95513 | 0 | N |
| Stock assessment | English sole | 0.81793 | 0 | N |
| Stock assessment | Greenstriped rockfish | 0.69398 | 2.08E-07 | Y |
| Stock assessment | Hake | 0.43865 | 0.001861568 | N |
| Stock assessment | Kelp greenling | -0.28021 | 0.149617781 | N |
| Stock assessment | Lingcod (California) | 0.93935 | 0 | N |
| Stock assessment | Lingod (Oregon & Washington) | 0.94542 | 0 | N |
| Stock assessment | Longnose skate | 0.49039 | 4.69E-05 | Y |
| Stock assessment | Longspine thornyhead | 0.80843 | 7.01E-08 | N |
| Stock assessment | Pacific mackerel | 0.26858 | 0.042765981 | N |
| Stock assessment | Pacific ocean perch | 0.42487 | 0.001466928 | N |
| Stock assessment | Petrale sole | 0.61738 | 2.64E-08 | N |
| Stock assessment | Sablefish | 0.8646 | 0 | Y |
| Stock assessment | Sardine | 0.66034 | 0.000193889 | N |
| Stock assessment | Scorpionfish | 0.78724 | 4.89E-10 | N |
| Stock assessment | Sheephead | 0.57088 | 5.40E-06 | N |
| Stock assessment | Shortbelly rockfish | 0.88416 | 0 | N |
| Stock assessment | Shortspine thornyhead | 0.88794 | 8.10E-09 | N |
| Stock assessment | Splitnose rockfish | 0.9223 | 0 | N |
| Stock assessment | Starry flounder (California) | 0.29898 | 0.054523788 | Y |
| Stock assessment | Starry flounder (Oregon) | 0.26858 | 0.065769607 | Y |
| Stock assessment | Widow Rockfish | 0.66901 | 3.07E-07 | Y |
| Stock assessment | Yelloweye rockfish | 0.90004 | 0 | Y |
| Stock assessment | Yellowtail rockfish | 0.49224 | 0.001147585 | Y |
| Atlantis | Adult shrimp | 0.99974 | 0 | N |
| Atlantis | Albacore | 0.86774 | 0 | N |
| Atlantis | Baleen whales | 0.79697 | 3.87E-14 | N |
| Atlantis | Benthic bacteria | 0.91352 | 0 | N |
| Atlantis | Benthic carnivores | 0.97019 | 0 | N |
| Atlantis | Benthic grazers | 1 | 0 | N |
| Atlantis | Canary rockfish | 0.91738 | 0 | N |
| Atlantis | Deep benthic filter feeders | 0.80176 | 6.67E-14 | N |
| Atlantis | Deep large rockfish | 0.75477 | 6.45E-13 | N |
| Atlantis | Deep megazoobenthos | 0.84456 | 0 | N |
| Atlantis | Deep miscellaneous fish | 0.7419 | 1.87E-11 | N |
| Atlantis | Deep vertical migrators | 0.54133 | 9.12E-06 | N |
| Atlantis | Deposit feeders | 0.85812 | 0 | N |
| Atlantis | Dover sole | 0.89444 | 0 | N |
| Atlantis | Gelatinous zooplankton | 0.83149 | 0 | N |
| Atlantis | Hake | 0.55794 | 3.43E-06 | N |
| Atlantis | Labile detritus | 0.80226 | 7.77E-16 | N |
| Atlantis | Large carnivorous zooplankton | 0.82023 | 0 | N |
| Atlantis | Large demersal predators | 0.82912 | 0 | Y |
| Atlantis | Large demersal sharks | 0.9429 | 0 | Y |
| Atlantis | Large flatfish | 0.8264 | 0 | N |
| Atlantis | Large megazoobenthos | 0.98654 | 0 | Y |
| Atlantis | Large phytoplankton | 0.66734 | 6.03E-09 | N |
| Atlantis | Large planktivorous fish | 0.52063 | 4.52E-05 | N |
| Atlantis | Longnose skate | 0.76739 | 1.32E-13 | Y |
| Atlantis | Longspine thornyhead | 0.81862 | 2.22E-16 | N |
| Atlantis | Meiobenthos | 0.98637 | 0 | N |
| Atlantis | Mesozooplankton | 0.81762 | 1.11E-16 | Y |
| Atlantis | Microalgae | 0.85003 | 0 | N |
| Atlantis | Microzooplankton | 0.64592 | 8.23E-09 | Y |
| Atlantis | Midwater rockfish | 0.90749 | 0 | N |
| Atlantis | Migrating seabirds | 0.99963 | 0 | N |
| Atlantis | Miscellaneous nearshore fish | 0.93056 | 0 | N |
| Atlantis | Miscellaneous pelagic sharks | 0.95051 | 0 | N |
| Atlantis | Nearshore fish | 0.99943 | 0 | N |
| Atlantis | Other benthic filter feeders | 1 | 0 | N |
| Atlantis | Pelagic bacteria | 0.84974 | 0 | N |
| Atlantis | Pinnipeds | 0.9719 | 0 | N |
| Atlantis | Piscivorous seabirds | 0.99744 | 0 | N |
| Atlantis | Planktivorous seabirds | 0.61366 | 2.16E-07 | N |
| Atlantis | Refractory detritus | 0.99333 | 0 | N |
| Atlantis | Sablefish | 0.88347 | 0 | Y |
| Atlantis | Salmon | 0.99041 | 0 | N |
| Atlantis | Sea otter | 0.99467 | 0 | N |
| Atlantis | Seagrass | 0.99996 | 0 | N |
| Atlantis | Shallow benthic filter feeders | 0.95774 | 0 | N |
| Atlantis | Shallow large rockfish | 0.8969 | 0 | N |
| Atlantis | Shallow small rockfish | 0.99742 | 0 | N |
| Atlantis | Shortbelly Rockfish | 0.60342 | 2.36E-07 | Y |
| Atlantis | Small cetaceans | 0.90547 | 0 | N |
| Atlantis | Small demersal sharks | 0.96096 | 0 | Y |
| Atlantis | Small flatfish | 0.8365 | 2.22E-16 | N |
| Atlantis | Small megazoobenthos | 0.98165 | 0 | N |
| Atlantis | Small phytoplankton | 0.83923 | 0 | N |
| Atlantis | Small planktivorous fish | 0.23239 | 0.047082618 | N |
| Atlantis | Squid | 0.99934 | 0 | N |
| Atlantis | Toothed whales | 0.99957 | 0 | N |
| Atlantis | Transient orca | 0.99789 | 0 | N |
| Atlantis | Yelloweye Rockfish | 0.96555 | 0 | N |
