# Supplementary material for: Stock assessment and end-to-end ecosystem models alter dynamics of fisheries data
Source: PLoS One. 2017 Feb 15;12(2):e0171644. doi: 10.1371/journal.pone.0171644 (PMC5310756; doi:10.1371/journal.pone.0171644)
Supplement: S2 Table — (DOCX) [file pone.0171644.s002.docx]

**S2 Table. Embedding dimension (E) results for each data type.**

| **Data/Model** | **Type** | **# Time series** | **E** |
| --- | --- | --- | --- |
| **Data** | Landings | 49 | 4.22±2.96 |
| **Data** | Abundance survey | 23 | 3.04±1.72 |
| **Model output** | Stock assessment | 36 | 5.14±2.91 |
| **Model output** | Stock as. + noise | 3600 | 4.16±2.69 |
| **Model output** | Atlantis | 59 | 5.15±2.85 |
| **Model output** | Atlantis + noise | 5900 | 4.27±2.47 |
