# Supplementary material for: Stock assessment and end-to-end ecosystem models alter dynamics of fisheries data
Source: PLoS One. 2017 Feb 15;12(2):e0171644. doi: 10.1371/journal.pone.0171644 (PMC5310756; doi:10.1371/journal.pone.0171644)
Supplement: S1 File — (DOCX) [file pone.0171644.s003.docx]

**S1 File (Supplemental Material)**

**Theory**

The forecasting methods described in the main text are based on Takens' theorem of lagged coordinates (Takens 1981). Takens’ theorem states that using time-lagged coordinates as a forecasting tool preserves the underlying dynamics of the system (Takens 1981). Lagged coordinates enable the modeler to obtain a shadow image of the system attractor. The proper number of dimensions will reveal the dynamics of the trajectories, but the modeler does not know how many dimensions are appropriate for the given data set (Sugihara and May 1990). For example, a time series of bluefin tuna may appear completely random because it is a one-dimensional picture of a system that in reality exists in more than one dimension (Sugihara and May 1990). In theory, if one can reconstruct the shape of the system attractor using Takens' embedding theorem in the proper number of dimensions, a seemingly random time series can become predictable.

As a classic example, S1 Fig. exhibits the Lorenz attractor (Lorenz 1963) in X-Y-Z coordinates.

**S1 Fig. Lorenz attractor in X-Y-Z coordinates, σ=10, b=8/3, r=26.**

The driving equations are:

ẋ=σ(y-x) ẏ = rx-y-xz ż=xy-bz

Where σ, b, and r are constants.
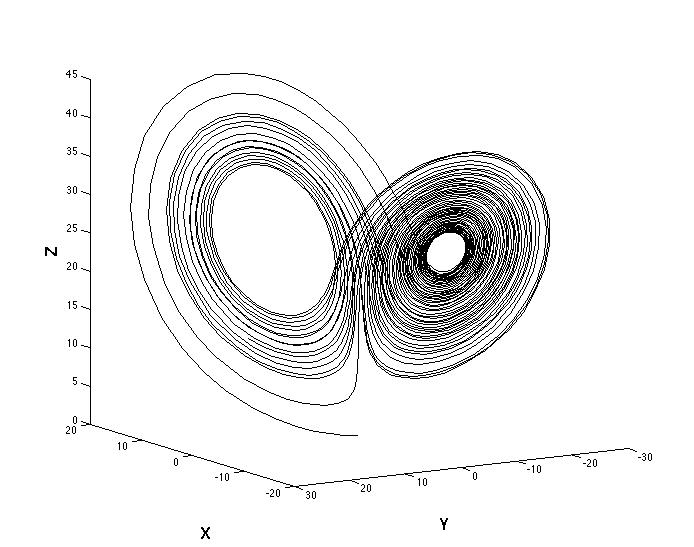

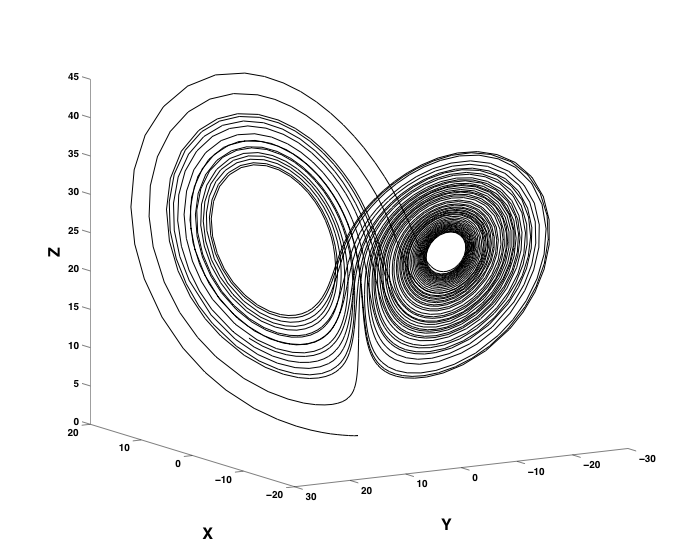

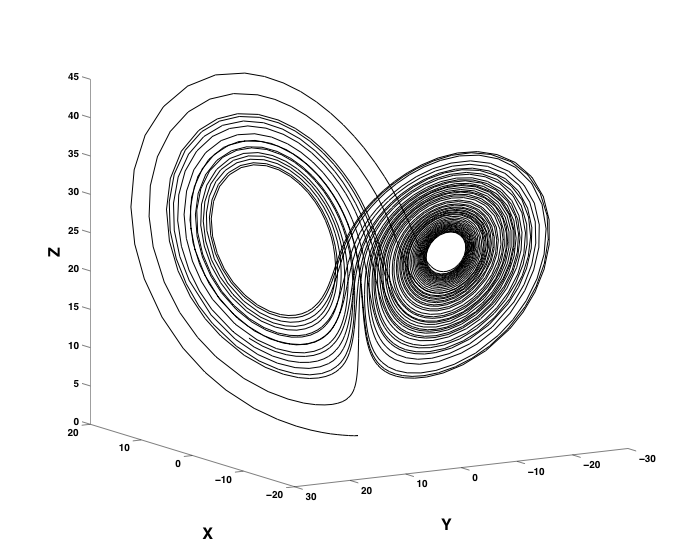
In one dimension, the dynamics of this three-dimensional system would be unresolvable, and in a one-dimensional graph, the dynamics may even appear to be stochastic. However, the boundaries of movement as defined by the attractor are well defined in the proper number of dimensions. If this attractor represented the biomass of a species, its form and the number of dimensions in which it resides can help identify the number and identity of environmental or anthropogenic factors that direct its dynamics.

**References**

Lorenz EN. 1963. Deterministic nonperiodic flow. J Atmos Sci. 1963; 20: 130-141.

Sugihara G., May RM. Nonlinear forecasting as a way of distinguishing chaos from measurement error in a time series. Nature. 1990; 344: 734-741.
